# HCV Ab titer and ALT level indicate occult hepatitis C virus infection in treatment-naive HCV Ab-positive and HCV Ab-negative patients: a 3-year prospective cohort study

**DOI:** 10.1128/spectrum.02922-24

**Published:** 2025-06-24

**Authors:** Ming-Ling Chang, Wei-Ting Chen, Li-Heng Pao, Cheng-Hsun Chiu, Rong-Nan Chien

**Affiliations:** 1 Division of Hepatology, Department of Gastroenterology and Hepatology, Chang Gung Memorial Hospital at Linkou 665228, Taoyuan, Taiwan; 2 Department of Medicine, College of Medicine, Chang Gung University 71589, Taoyuan, Taiwan; 3 Graduate Institute of Health Industry Technology, Research Center for Food and Cosmetic Safety, and Research Center for Chinese Herbal Medicine, College of Human Ecology, Chang Gung University of Science and Technology 684947, Taoyuan, Taiwan; 4 Molecular Infectious Disease Research Center, Chang Gung Memorial Hospital at Linkou 38014, Taoyuan, Taiwan; 5 Division of Pediatric Infectious Diseases, Department of Pediatrics, Chang Gung Memorial Hospital at Linkou 38014, Taoyuan, Taiwan; Indian Institute of Science, Bangalore, Karnataka, India

**Keywords:** OCI, HCV Ab, treatment-naive, cardiovascular, cryptogenic hepatitis

## Abstract

The phenotype of occult hepatitis C virus (HCV) infection (OCI) in anti-HCV treatment-naive patients remains elusive. A prospective 3-year cohort study of 218 anti-HCV treatment-naive patients was conducted. OCI was defined as positive HCV RNA in peripheral blood mononuclear cells (PBMCs) of patients with negative serum HCV RNA. Among the 128 HCV antibody (Ab)-positive patients, 15 (11.7%) had OCI; higher HCV Ab titers were noted in patients with OCI than in those without OCI (84.25 ± 64.76 vs 46.60 ± 46.07 ng/mL, *P* = 0.006), and the HCV Ab titer was a marker of OCI (cutoff: >53.2 ng/mL). The 3-year cumulative incidence (CI) of cardiovascular events was greater in patients with OCI than in those without OCI (84% vs 46.4%, *P* = 0.043). Among the 90 HCV Ab-negative patients, 5 (5.6%) had OCI; the alanine aminotransferase (ALT) levels were greater in patients with OCI than in those without OCI (114.4 ± 71.6 vs 67.2 ± 87.4 U/L, *P* = 0.035). A greater CI of autoimmune disease was noted in patients with OCI than in those without OCI (20% vs 1.2%, *P* = 0.006). Among anti-HCV treatment-naive patients, the prevalence rates of OCI were 11.7% in HCV Ab-positive patients and 5.6% in HCV Ab-negative patients. High HCV Ab titers in HCV Ab-positive patients require caution regarding OCI and cardiovascular events, and cryptogenic hepatitis warrants suspicion of OCI and autoimmune diseases.

**IMPORTANCE:**

Among anti-HCV treatment-naive patients, the prevalence rates of OCI were 11.7% in HCV Ab-positive patients and 5.6% in HCV Ab-negative patients. High HCV Ab titers (cutoff: >53.2 ng/mL) in HCV Ab-positive patients require caution regarding OCI and cardiovascular events, and cryptogenic hepatitis warrants suspicion of OCI and autoimmune diseases.

## INTRODUCTION

Hepatitis C virus (HCV) is a human pathogen responsible for chronic liver diseases, infecting approximately 71.1 million individuals worldwide (1). Hepatic complications, such as hepatocellular carcinoma (HCC) (2), cirrhosis and steatotic liver disease (3), and extrahepatic complications, including mixed cryoglobulinemia (4), extrahepatic cancers (5), cardiometabolic events (6), and autoimmune diseases (7), are associated with chronic hepatitis C (CHC). Like CHC, occult HCV infection (OCI), defined as the presence of the HCV genome in either liver tissue or peripheral blood mononuclear cells (PBMCs), despite constant negative results from tests for HCV RNA in serum (8), seems to significantly affect patient prognosis. For example, OCI has been associated with increased mean alanine aminotransferase (ALT) levels and mortality among patients who underwent hemodialysis (9) and with susceptibility to hepatic decompensation and post-therapy HCC among CHC patients who developed sustained virological response (SVR) following direct-acting antiviral (DAA) therapy (10). There are the following two types of OCIs: seronegative [anti-HCV antibody (Ab)-negative and serum HCV RNA-negative] (11, 12) and seropositive (anti-HCV Ab-positive and serum HCV RNA-negative). The latter is also called secondary OCI (13), which represents either OCI in patients who were assumed to have spontaneous HCV clearance without any prior HCV treatment or OCI in CHC patients who acquired SVRs following anti-HCV therapies. OCI indicates ongoing viral replication, and the percentage of HCV replication in the PBMCs of OCI patients is similar to that in the PBMCs of CHC patients (13). The risk of intrafamily HCV transmission is even greater in OCI patients than in CHC patients, as the prevalence of HCV infection among family members of OCI patients (9.8%) is greater than that among CHC patients (3.4%) (14). Both seronegative and seropositive patients are assumed to have no HCV infection, neither patient group would receive anti-HCV therapies, and the emergence of OCI in these patients hinders HCV elimination. Although the gold standard for diagnosing OCI is liver biopsy, examination of PBMCs may be a reliable, safer alternative method for diagnosing OCI (14), as HCV-RNA is detected in the PBMCs of 92.3% of patients with intrahepatic HCV-RNA (15), and serial testing of PBMCs helps increase the detection rate of OCI (16). Although some of these data come from China (17), most of the available OCI information has come from studies based in Western countries (13). OCI information remains elusive, especially in Taiwan, an Asian country endemic for hepatitis C (4).

Accordingly, we aimed to elucidate the prevalence rates and associated phenotypes of OCI, diagnosed by positive HCV RNA in PBMCs on at least two occasions among anti-HCV treatment-naive patients with either seronegative or seropositive OCI, by conducting a prospective study to evaluate the presence of OCI and various complications every 3–6 months during follow-up.

## MATERIALS AND METHODS

### Patients

**Fig 1:**
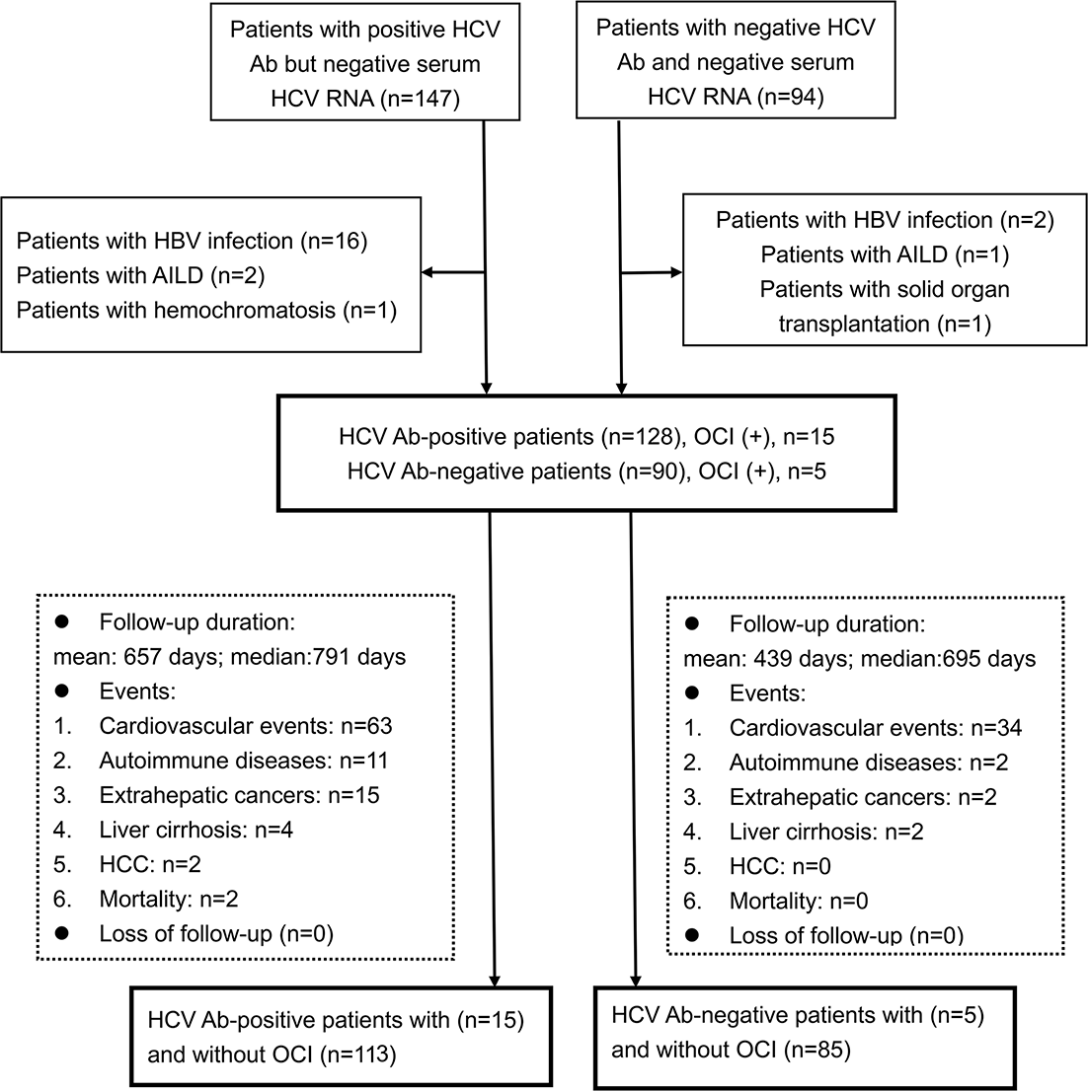
Flowchart of patient enrollment. HCV, hepatitis C virus; HBV, hepatitis B virus infection; AILD, autoimmune liver disease; OCI, occult HCV infection; HCC, hepatocellular carcinoma.

The study included HCV Ab-positive and HCV Ab-negative patients. The former group was composed of anti-HCV treatment-naive patients with HCV Abs but undetectable serum HCV RNA (on at least two occasions, 6 months apart). The latter group was composed of HCV Ab-negative patients with undetectable serum HCV-RNA (at least two occasions, 6 months apart). Subjects with human immunodeficiency virus or hepatitis B virus infection, hemochromatosis, autoimmune liver diseases, or a solid organ transplant at baseline were excluded. The flowchart of patient enrollment and follow-up is summarized in Fig. 1.

### Study design

Patients were consecutively recruited from a Taiwan tertiary referral center between July 2020 and October 2023. Several factors, including sex, age, body mass index (BMI), HCV Ab (Roche Elecsys Anti-HCV II, ZUG, Switzerland), HCV RNA (Roche Cobas AmpliPrep/COBAS TaqMan HCV Test 2.0, ZUG, Switzerland), HCV genotype (Roche Cobas GT, ZUG, Switzerland), aspartate transaminase (AST), alanine aminotransferase (ALT), alpha-fetoprotein (AFP), cryoglobulins, presence of hepatic cirrhosis, estimated glomerular filtration rate (eGFR), homeostatic model assessment for insulin resistance (HOMA-IR), total cholesterol (TC), triglycerides (TGs), and mixed cryoglobulinemia, were assessed and recorded at baseline. Biochemical tests were performed at the clinical pathology laboratories of the hospital using routine automated techniques, and cryoglobulins were measured using the double immunodiffusion method (18).

### Occult HCV infection surveys

PBMCs from the enrolled patients were isolated by Ficoll Paque PLUS (Pharmacia Biotech, Quebec, Canada) with gradient fractionation. HCV RNA was extracted from 1 × 10^7^ PBMCs using TRIzol (Invitrogen) following the manufacturer’s instructions (19) and was assessed using an HCV RNA (Roche) kit. Given that OCI can be transient, with a trend toward a decrease in the HCV viral load to levels undetectable by conventional methods after 12-18 months (20), HCV RNA from PBMCs was assessed for each patient on at least 2 occasions 6 months apart. The viral load of each person was taken as the highest value of all the positive results from each measurement.

### Outcomes

Cardiovascular events were defined as ischemic heart disease, coronary revascularization, stroke, heart failure, cardiac arrest, or cardiovascular death and were identified using the International Classification of Diseases, Ninth Revision, Clinical Modification (ICD-9, CM) by patient reports and confirmed by a review of medical records/registries. Cancers were diagnosed based on pathology and confirmed by specialists for each primary cancer, and the diagnosis and stage of each cancer followed the National Cancer Registry (21, 22). Hepatic events included cirrhosis and HCC. The diagnosis of liver cirrhosis and HCC (23) was made as described previously. Patients were also assessed for autoimmune diseases, including Sjögren syndrome, systemic lupus erythematosus, systemic sclerosis, rheumatoid arthritis, autoimmune thyroid disease, psoriasis, ulcerative colitis, Crohn’s disease, and autoimmune liver diseases (7), during a 3-year follow-up.

### Statistics

All statistical analyses were performed using either Statistical Product and Service Solutions (SPSS ver. 21.0, SPSS, Inc., Chicago, IL, USA) or MedCalc (MedCalc ver. 12.4, MedCalc Software Corp., ME, USA) software. Continuous variables are presented as the means ± standard deviations (SDs), and categorical variables are presented as the frequencies and percentages. To compare different variables in different groups, continuous variables were analyzed using Student’s *t* tests, whereas categorical variables were analyzed using the chi-squared tests or Fisher’s exact tests, as appropriate. Nonparametric analyses were performed when indicated. Multivariate regression models were used to assess the association between dependent and independent factors by adjusting for all independent variables with *P* < 0.05 in univariate analyses. Kaplan‒Meier or univariate Cox regression analyses were used to assess the relationships between various variables and patient events. Receiver-operating characteristic (ROC) analyses were performed to evaluate whether the independent variables were significant predictors of the dependent variables. The Youden index was calculated to identify the best cutoff values of the independent variables from the coordinate points of the ROC curves. Genotype association tests were performed using logistic regression analyses with additive models. Statistical significance was defined at the 5% level based on two-tailed tests of the null hypothesis.

## RESULTS

### Baseline characteristics

**Fig 2:**
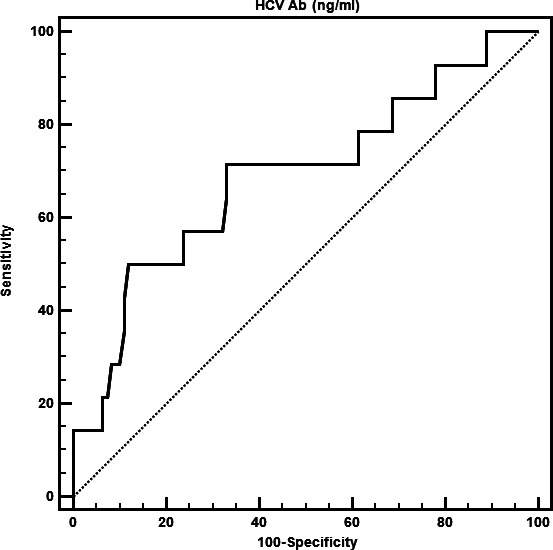
The area under the receiver-operating characteristic curve for the ability of HCV Abs to predict OCI among HCV-positive patients.

At baseline, a total of 218 patients (128 anti-HCV treatment-naive patients who were positive for HCV Abs but negative for serum HCV RNA and 90 patients who were negative for HCV Abs and serum HCV RNA) were enrolled (Table 1). Among the 128 HCV Ab-positive patients, 46 (35.9%) were males, and 15 (11.7%) had OCI; the mean and median ages of these patients were 60.5 and 62.0 years, respectively. Among the 90 HCV Ab-negative patients, 49 (54.4%) were males, 5 (5.6%) had OCI, and the mean and median ages were 49.1 and 50.0 years, respectively (Table 2). The HCV Ab-positive patients were older, had lower BMIs and ALT levels, and had a lower rate of fatty liver than the HCV Ab-negative patients (Table 1). Among the 15 HCV Ab-positive OCI patients, 5 (33.3%) were males, with mean and median ages of 58.9 and 60.0 years, respectively (Table 2); the mean and median viral loads were 1,046 IU in 10^7^ PBMCs and 28.38 IU in 10^7^ PBMCs, respectively, and the viral loads ranged from 5.45 IU in 10^7^ PBMCs to 14,883 IU in 10^7^ PBMCs. Among the 15 OCI patients, 6 (40%) had only one positive result for HCV RNA in PBMCs among 2–3 surveys (the highest variation in viral load among the same individual with several detections was 0 ~ 14,883 IU in 10^7^ PBMCs). Among the five HCV Ab-negative OCI patients, two (40%) were males, with mean and median ages of 42.2 and 46.0 years, respectively; their mean and median viral loads were 2,356 IU in 10^7^ PBMCs and 111.7 IU in 10^7^ PBMCs, respectively, and their viral loads ranged from 29.04 IU in 10^7^ PBMCs to 7,100 IU in 10^7^ PBMCs. Among the five OCI patients, two (40%) had only one positive result for HCV RNA in PBMCs according to two or three tests (the highest variation in the viral load in the same individual with several detections was 0–7,100 IU in 10^7^ PBMCs). There was no significant difference in viral load between HCV Ab-positive and HCV Ab-negative OCI patients. Genotypes were not assessable in any of the OCI patients. All 15 HCV Ab-positive OCI patients had a normal liver function, whereas all five HCV Ab-negative OCI patients had an abnormal liver function. Specifically, among HCV Ab-positive patients, the ALT levels in those with and without OCI ranged from 6 U/L to 33 U/L (normal <36 U/L) and from 7 U/L to 97 U/L, respectively. Among the HCV Ab-negative patients, the ALT levels in those with and without OCI ranged from 49 U/L to 223 U/L and from 7 U/L to 97 U/L, respectively. Regardless of HCV Ab positivity, most of the investigated baseline factors were comparable between patients with and without OCIs (Table 2). However, among the HCV Ab-positive patients, higher HCV titers were noted in patients with OCI than in those without OCI. The cutoff titer of HCV Ab for predicting OCI was >53.2 ng/mL (sensitivity: 71.4%; specificity: 67.0%; area under the ROC curve (AUROC): 0.701; 95% CI: 0.600–0.770; *P* = 0.0254) among the HCV-positive patients (Fig. 2); among the HCV Ab-negative patients, higher ALT levels were noted in patients with OCI than in those without OCI (Table 2).

**TABLE 1:** Comparison of baseline characteristics between patients with and without HCV Abs^*a*^

Characteristic	HCV Abs (+) (*n* = 128)	HCV Abs (−) (*n* = 90)	*P* value
Males, *n* (%)	46 (35.9)	49 (54)	0.007
Age (years)	60.51 ± 12.62	49.07 ± 12.76	<0.001
BMI (kg/m²)	25.84 ± 4.21	27.75 ± 4.20	0.001
HCV Abs (ng/ml)	50.88 ± 49.16	NA	NA
AST (U/L)	24.02 ± 9.44	42.15 ± 6.84	0.015
ALT (U/L)	24.69 ± 15.91	69.84 ± 86.00	<0.001
AFP (ng/ml)	3.07 ± 1.84	2.94 ± 1.14	0.955
eGFR (ml/min/1.73 m^2^)	95.99 ± 34.62	98.15 ± 22.39	0.615
TC (mg/dL)	191.15 ± 37.27	199.22 ± 37.21	0.038
TGs (mg/dL)	140.12 ± 95.74	158.37 ± 92.67	0.274
HOMA-IR	2.47 ± 2.93	2.66 ± 1.84	0.857
FIB-4	1.45 ± 7.89	1.11 ± 2.13	0.123
Fatty liver, *n* (%)	74 (57.8)	86 (95.6)	<0.001
Cirrhosis, *n* (%)	4 (3.1)	2 (2.2)	0.69
MC, *n* (%)	29 (22.7)	11 (12.2)	0.209

^*a*^ BMI, body mass index; NA, not assessable; AST, aspartate aminotransferase; ALT, alanine aminotransferase; AFP, alpha-fetoprotein; eGFR, estimated glomerular filtration rate; TC, total cholesterol; TGs, triglycerides; HOMA-IR, homeostasis model assessment of insulin resistance; FIB-4, fibrosis-4 score; MC, mixed cryoglobulinemia**.**

**TABLE 2:** Comparison of baseline characteristics between patients with and without OCI^*a*^

Characteristic	HCV Ab-positive patients (*n* = 128)			HCV Ab-negative patients (*n* = 90)		
	OCI (+) (*n* = 15)	OCI (-) (*n* = 113)	*P* value	OCI (+) (*n* = 5)	OCI (-) (*n* = 85)	*P* value
Males, *n* (%)	5 (33.3)	41(36.6)	0.553	2 (40.0)	47 (55.3)	0.415
Age (years)	58.71 ± 16.41	61.21 ± 12.27	0.494	42.20 ± 15.16	49.75 ± 12.42	0.194
BMI (kg/m²)	25.25 ± 3.08	25.91 ± 4.42	0.589	27.22 ± 5.10	27.79 ± 4.15	0.767
HCV Abs (ng/mL)	84.25 ± 60.76	48.38 ± 46.02	0.010	NA	NA	NA
AST (U/L)	26.77 ± 10.80	23.92 ± 9.38	0.313	42.00 ± 15.22	40.19 ± 62.78	0.949
ALT (U/L)	16.77 ± 8.07	24.95 ± 15.58	0.066	114.60 ± 71.67	64.01 ± 85.07	0.035
AFP (ng/mL)	2.60 ± 0.50	3.07 ± 1.96	0.428	2.98 ± 1.37	3.02 ± 1.19	0.948
eGFR (mL/min/1.73 m^2^)	87.90 ± 36.91	97.19 ± 35.69	0.425	114.00 ± 33.27	97.34 ± 20.78	0.139
TC (mg/dL)	203.67 ± 30.84	187.24 ± 37.54	0.150	192.80 ± 50.72	200.95 ± 35.94	0.632
TGs (mg/dL)	192.92 ± 189.65	134.65 ± 78.48	0.314	170.00 ± 175.58	155.96 ± 85.29	0.868
HOMA-IR	2.99 ± 3.77	2.45 ± 2.92	0.569	3.16 ± 1.81	2.57 ± 1.80	0.522
FIB-4	1.39 ± 0.63	1.49 ± 0.82	0.728	0.69 ± 0.32	1.13 ± 2.18	0.689
Fatty liver, *n* (%)	6 (40)	68 (60.2)	0.114	5 (100)	81 (95)	0.792
Cirrhosis, *n* (%)	1 (6.7)	3 (2.7)	0.396	0 (0)	2 (2.4)	0.891
MC, *n* (%)	5 (33.3)	24 (21.2)	0.206	2 (40)	11 (12.9)	0.557

^*a*^ OCI, occult HCV infection; BMI, body mass index; NA, not assessable; AST, aspartate aminotransferase; ALT, alanine aminotransferase; AFP, alpha-fetoprotein; eGFR, estimated glomerular filtration rate; TC, total cholesterol; TGs, triglycerides; HOMA-IR, homeostasis model assessment of insulin resistance; FIB-4, fibrosis-4 score; MC, mixed cryoglobulinemia.

### Outcomes

The follow-up durations and main outcomes are shown in Fig. 1. Among the 128 HCV Ab-positive patients, most of their extrahepatic cancers (*n* = 15) were colon cancers (*n* = 3, 20%) (Table S1). Two (1.6%) patients died due to terminal lung cancer or sepsis. Neither of the two patients who died had OCI. Specifically, compared with the HCV Ab-positive patients without OCI, those with OCI had a greater cumulative incidence of cardiovascular events (84% vs 46.4%, *P* = 0.043) (Fig. S1A), but no significant differences in the cumulative incidences of autoimmune diseases (6.7% vs 15.6%, *p*=0.762) (Fig. S1B), extrahepatic cancer (6.7% vs 19.6%, *p*=0.352) (Fig. S1C), liver cirrhosis (6.7% vs 2.7%, *p*=0.403) (Fig. S1D), HCC (0% vs 3.6%, *P* = 0.549) (Fig. S1E), and mortality (0% vs 4.4%, *P* = 0.515) (Fig. S1F) were noted between these two groups. Multivariate analysis revealed that only age was independently associated with cardiovascular event development (Table 3). Among the 90 HCV Ab-negative patients, none had HCC. One patient developed lung cancer, and one developed thyroid cancer. A greater cumulative incidence of autoimmune disease was noted in the HCV Ab-negative patients with OCI than in those without OCI (20% vs 1.2%, *P* = 0.006) (Fig. S2A), but these two groups had similar cumulative incidences of cardiovascular events (33.3% vs 38%, *P*=0.819) (Fig. S2B), extrahepatic cancers (0% vs 9.7%, *P* = 0.564) (Fig. S2C), and liver cirrhosis (0% vs 2.2%, *P* = 0.717) (Fig. S2D). No independent factors were noted among the investigated baseline factors for autoimmune diseases in HCV Ab-negative patients. Interestingly, higher cumulative incidences of extrahepatic cancer (17.5% vs 7.3%, *P* = 0.015) (Fig. S3A) and autoimmune diseases (14.4% vs 2.2%, *p*=0.043) (Fig. S3B) were noted in the HCV Ab-positive patients than in the HCV Ab-negative patients. Similar cumulative incidences of cardiovascular events (62% vs 37.8%, *P* = 0.118) (Fig. S3C), HCC (3% vs 0%, *P* = 0.32) (Fig. S3D), and liver cirrhosis (3.1% vs 2.2%, *p*=0.689) (Fig. S3E) were noted between the HCV Ab-positive and HCV Ab-negative patients.

**TABLE 3:** Cox regression for baseline factors of cardiovascular events in HCV Ab-positive patients^*a*^

Variable	Univariate	
	Crude HR (95% CI)	*P* value
Age (years)	1.029 (1.007–1.052)	0.010#
BMI (kg/m²)	1.024 (0.967–1.085)	0.413
HCV Abs (ng/mL)	1.000 (0.995–1.005)	0.936
AST (U/L)	1.015 (0.992–1.035)	0.198
ALT (U/L)	1.005 (0.989–1.021)	0.580
AFP (ng/mL)	0.991 (0.849–1.156)	0.908
eGFR (mL/min/1.73 m^2^)	0.996 (0.987–1.004)	0.327
Total cholesterol (mg/dL)	0.995 (0.987–1.002)	0.186
TGs (mg/dL)	1.000 (0.997–1.003)	0.911
HOMA-IR	1.058 (0.985–1.137)	0.124
FIB-4	1.216 (0.886–1.670)	0.227
Fatty liver (yes)	1.143 (0.689–1.898)	0.604
Cirrhosis (yes)	1.59 (0.489–5.072)	0.433
Mixed cryoglobulinemia (yes)	1.001 (0.937–1.069)	0.975
OCI	1.514 (0.784–2.92)	0.216

^*a*^ HR, hazard ratio; CI, confidence interval; BMI, body mass index; NA, not assessable; AST, aspartate aminotransferase; ALT, alanine aminotransferase; AFP, alpha-fetoprotein; eGFR, estimated glomerular filtration rate; TC, total cholesterol; TGs, triglycerides; HOMA-IR, homeostasis model assessment of insulin resistance; FIB-4, fibrosis-4 score; MC, mixed cryoglobulinemia; OCI, occult HCV infection; #, only one factor (age) was identified as a significant factor by univariate analysis, and thus multivariate analysis was not conducted.

## DISCUSSION

Among the general population, OCI rates have been reported to range from 2.2% to 3.3%, and OCI rates reach 57% among individuals with abnormal liver function (13). The OCI rates in HCV Ab-positive and HCV Ab-negative patients were 11.7% and 5.6%, respectively, which are within the reported range (2.2%–57%) (13). Using *in situ* hybridization assays on liver biopsies with RNAscope, the OCI rate of treatment-naïve HCV Ab-positive patients in China was reported to be 6.7% (15), which is lower than that (11.7%) of our treatment-naive HCV Ab-positive patients; thus, we are confident that PBMCs are a feasible alternative to liver tissue for detecting OCI. Regardless of HCV Ab positivity, 40% of the OCI patients had only one detection of HCV RNA in 2–3 surveys of PBMCs, and the viral loads detected in different surveys were quite diverse for the same individuals. This finding was consistent with the observation that HCV viremia levels fluctuated during the follow-up period (16) and explained why the reported OCI incidence rates are inconsistent, ranging from 0% (24) to 55% (8).

Notably, among the HCV Ab-positive patients, all patients with OCI had normal ALT levels, whereas some of the patients without OCI had abnormal ALT levels. In these HCV Ab-positive OCI patients, the viral loads in the PBMCs were quite variable, ranging from 5.45 IU to 14,883 IU in 10^7^ PBMCs; the mean viral load of 1,046 IU in 10^7^ PBMCs (i.e., 104.6 IU/million PBMCs) seemed to be lower than that (357.4 ± 42.1 IU/million) reported among SVR patients with hepatic decompensation (10). Whether HCV in these HCV Ab-positive patients resides mostly in PBMCs with a low viral load and keeps the host liver indolent requires further investigation. In contrast, all the HCV Ab-negative OCI patients exhibited abnormal liver function. Notably, in HCV Ab-negative patients with elevated liver function test results, HCV RNA was detected in liver biopsy specimens from 74.2% of the patients (25), and the estimated frequency of OCI among patients displaying hepatic dysfunction was as high as 57% (26). Future studies are needed to determine whether OCI tends to elicit hepatic injury in patients who fail to produce HCV Abs in response to HCV infection. Given that the general CHC rate in Taiwan is only 3.3% (27), the OCI rate of 5.6% in HCV Ab-negative patients with abnormal liver function suggests the necessity to comprehensively investigate OCI among cryptogenic hepatitis patients in Taiwan to accelerate HCV elimination, as HCV RNA in PBMCs is infectious (19) and likely transmitted sexually (28).

Various prevalence rates of infection with some HCV genotypes, such as genotypes 1 (17, 29), 2 (17, 30), 3 (15, 19), and 6 a (17), have been reported in OCI patients; alternatively, OCI might be a universal phenomenon involving all genotypes (31). However, in the present study, no HCV genotype associated with OCI could be documented, and the viral loads might have been too low to identify the HCV genotype. Male sex and age above 30 years have been associated with the presence of OCI (25). However, in the present study, females accounted for the majority (over 60%) of OCI cases among both HCV Ab-positive and HCV Ab-negative patients, and no significant difference in age was noted between patients with and without OCI. Any ethnic factor accounting for this discrepancy awaits discovery in the future.

Fibrosis and necroinflammatory activity have been detected in the liver in up to 5% and 35% of OCI patients, respectively (31). However, all HCV Ab-positive patients with OCI had normal liver function, and the cumulative incidences of hepatic events were similar between the HCV Ab-positive patients with and without OCI. In addition to the low HCV load in PBMCs of the enrolled patients, as mentioned previously, further investigation is needed with respect to whether the presence of HCV in PBMCs of treatment-naive HCV Ab-positive patients suggests “residence selection” for HCV to cause negligible harm to the liver. Although all the HCV Ab-negative patients with OCI had higher ALT levels than their counterparts, no difference in the cumulative incidence of hepatic events was noted between the HCV Ab-negative patients with and without OCI, probably due to the small number (*n* = 5) of these OCI patients. Current evidence suggests that SVR in CHC patients reduces the incidence and mortality of cardiovascular disease and type 2 diabetes (3) and highlights the causal relationship between HCV infection and cardiovascular events. Although only age was an independent factor for cardiovascular events among HCV Ab-positive patients, given that demographic and metabolic profiles were comparable between HCV Ab-positive patients with and without OCI, the finding of higher cumulative incidences of cardiovascular events in patients with OCI than those without OCI might at least partially stem from OCI-related inflammation, particularly HCV residing in PBMCs, which might elicit local endothelial inflammation (3). Thus, among HCV Ab-positive patients, the presence of OCI indicates the necessity of tailored follow-up for emergent cardiovascular events. On the other hand, the link between HCV infection and autoimmune disease (6) is in line with the observations of a greater cumulative incidence of autoimmune diseases in HCV Ab-negative patients with OCI than in those without OCI and in more HCV Ab-positive patients than in HCV Ab-negative patients. In addition, a higher cumulative incidence of extrahepatic cancer was noted in HCV Ab-positive patients than in HCV Ab-negative patients, highlighting the oncogenic potential of HCV infection in many extrahepatic cancers (5) and their occasional irreversibility after HCV clearance. On the other hand, although OCI is a risk factor for lymphoma (28), only one HCV Ab-positive patient without OCI developed lymphoma in our cohort. Whether OCI has any role in lymphoma development requires further investigation.

There are several limitations to the current study. First, the total number of 218 cases might be too small to determine the general OCI prevalence in Taiwan, especially because some patients were HCV Ab-positive and the others were HCV Ab-negative. Second, given that all the patients were serum HCV RNA negative, and some were even HCV Ab negative, their PBMC HCV RNA levels are expected to be low, which might account for why the HCV genotype was not detectable. However, although the kit to test for PBMC HCV RNA included a negative control, the possibility of false-positive results cannot be excluded completely as the HCV genotype was not assessable. Third, although among the HCV Ab-negative patients, a higher cumulative incidence of autoimmune diseases was noted in patients with OCI than in those without OCI, the low number of cases (particularly, only five OCI patients) might lead to bias in the statistical results. Future large-scale cohort studies of both HCV Ab-positive and HCV-negative Taiwanese patients, with PBMC RNA tests performed via a qualitative real-time polymerase chain reaction assay and sophisticated molecular techniques, are needed to verify the results and elucidate the fundamental mechanisms underlying the findings described herein.

In summary, in 128 HCV Ab-positive patients and 90 HCV Ab-negative patients, the OCI rates were 11.7% and 5.6%, respectively. High HCV Ab titers (cutoff value: >53.2 ng/mL) and ALT levels indicate OCI in HCV Ab-positive and HCV Ab-negative patients, respectively. Special caution is needed for cardiovascular events in HCV Ab-positive patients with OCI and for autoimmune diseases in HCV Ab-negative patients with OCI.

## Data Availability

The data sets used and/or analyzed during the current study are available from the corresponding author upon reasonable request. De-identified data are available upon reasonable request, due to the amount and sensitivity of the data.

## SUPPLEMENTAL MATERIAL

Figure S1 spectrum.02922-24-s0001.tif Cumulative incidences of various outcomes of HCV-positive patients.

Figure S2 spectrum.02922-24-s0002.tif Cumulative incidences of various outcomes of HCV Ab-negative patients.

Figure S3 spectrum.02922-24-s0003.tif Cumulative incidences of various outcomes of all patients.

Supplemental legends spectrum.02922-24-s0004.docx Legends for Fig. S1 to S3.

Table S1 spectrum.02922-24-s0005.docx Extrahepatic cancers of HCV Ab-positive patients.

ASM does not own the copyrights to Supplemental Material that may be linked to, or accessed through, an article. The authors have granted ASM a non-exclusive, world-wide license to publish the Supplemental Material files. Please contact the corresponding author directly for reuse.
