# Supplementary figures and images for: HCV Ab titer and ALT level indicate occult hepatitis C virus infection in treatment-naive HCV Ab-positive and HCV Ab-negative patients: a 3-year prospective cohort study
Source: Microbiol Spectr. 2025 Jun 24;13(8):e02922-24. doi: 10.1128/spectrum.02922-24 (PMC12323646; doi:10.1128/spectrum.02922-24)

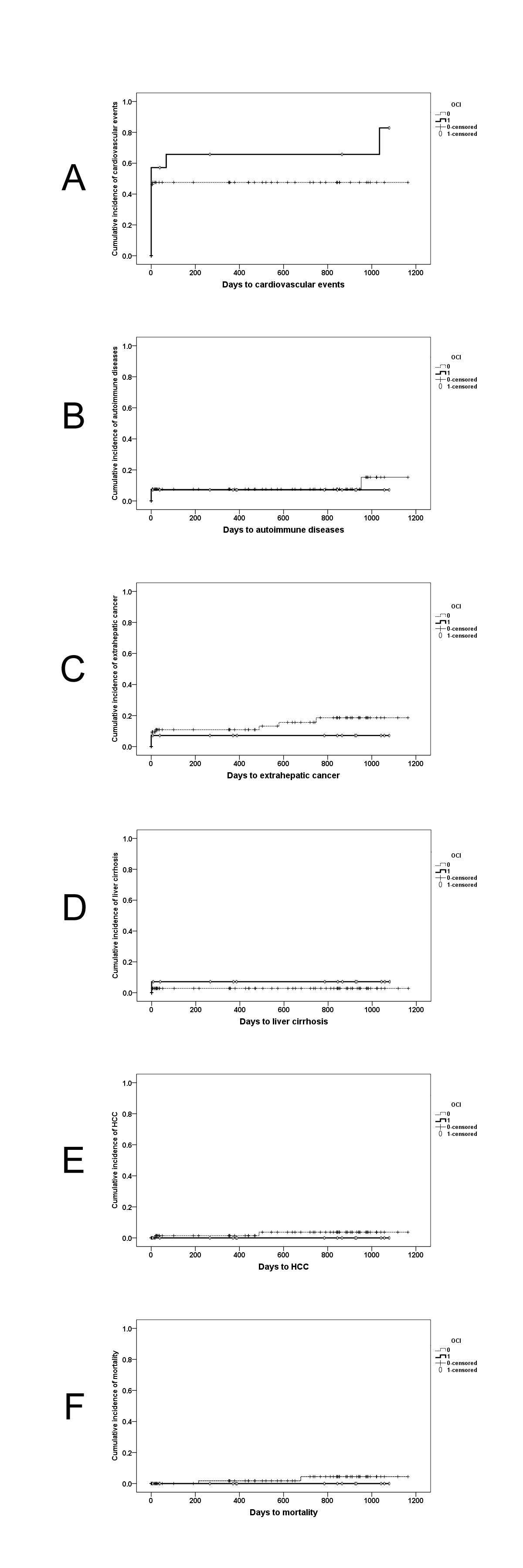

Supplement: Figure S1 — Cumulative incidences of various outcomes of HCV-positive patients. [file spectrum.02922-24-s0001.tif]

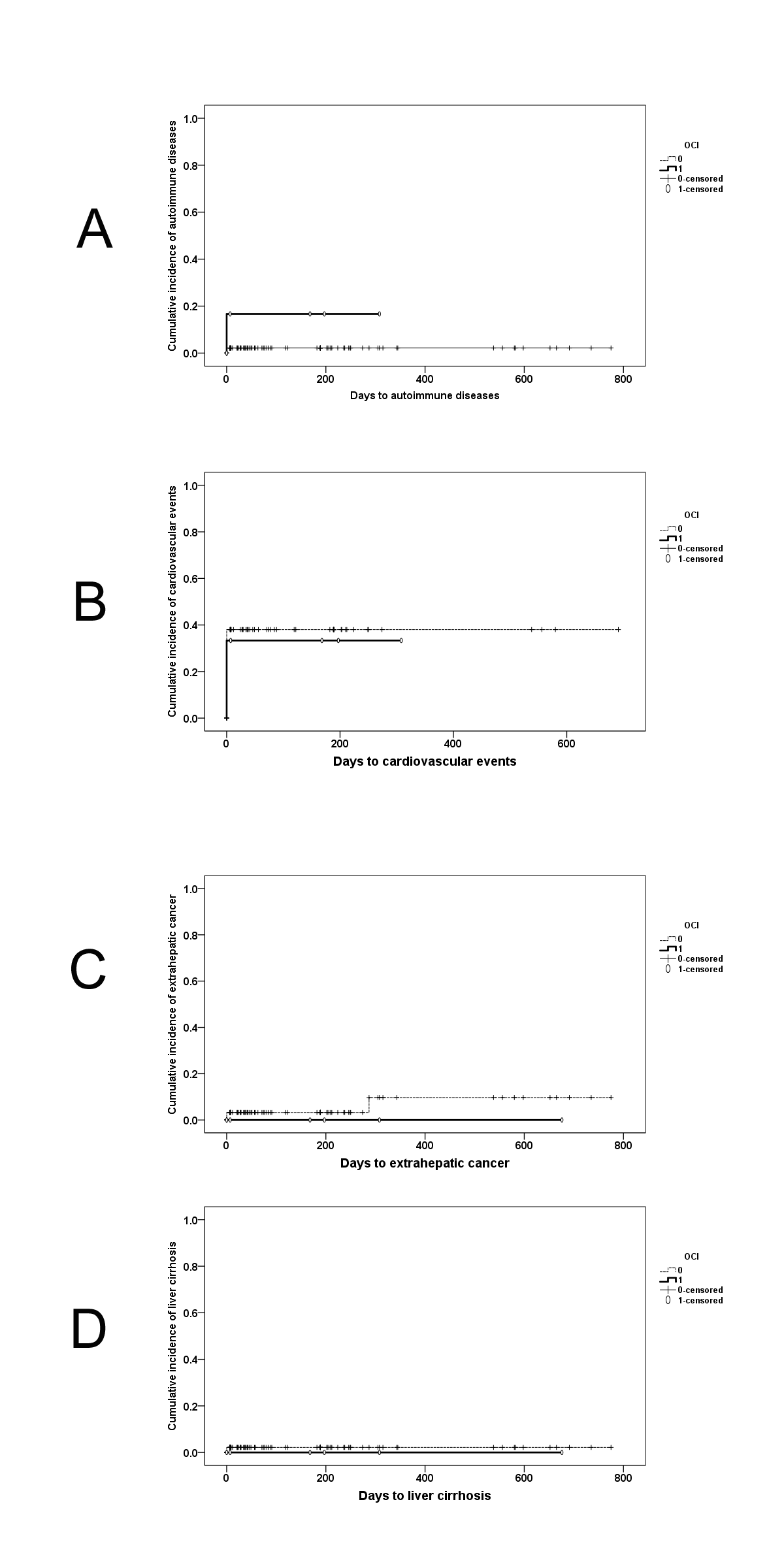

Supplement: Figure S2 — Cumulative incidences of various outcomes of HCV Ab-negative patients. [file spectrum.02922-24-s0002.tif]

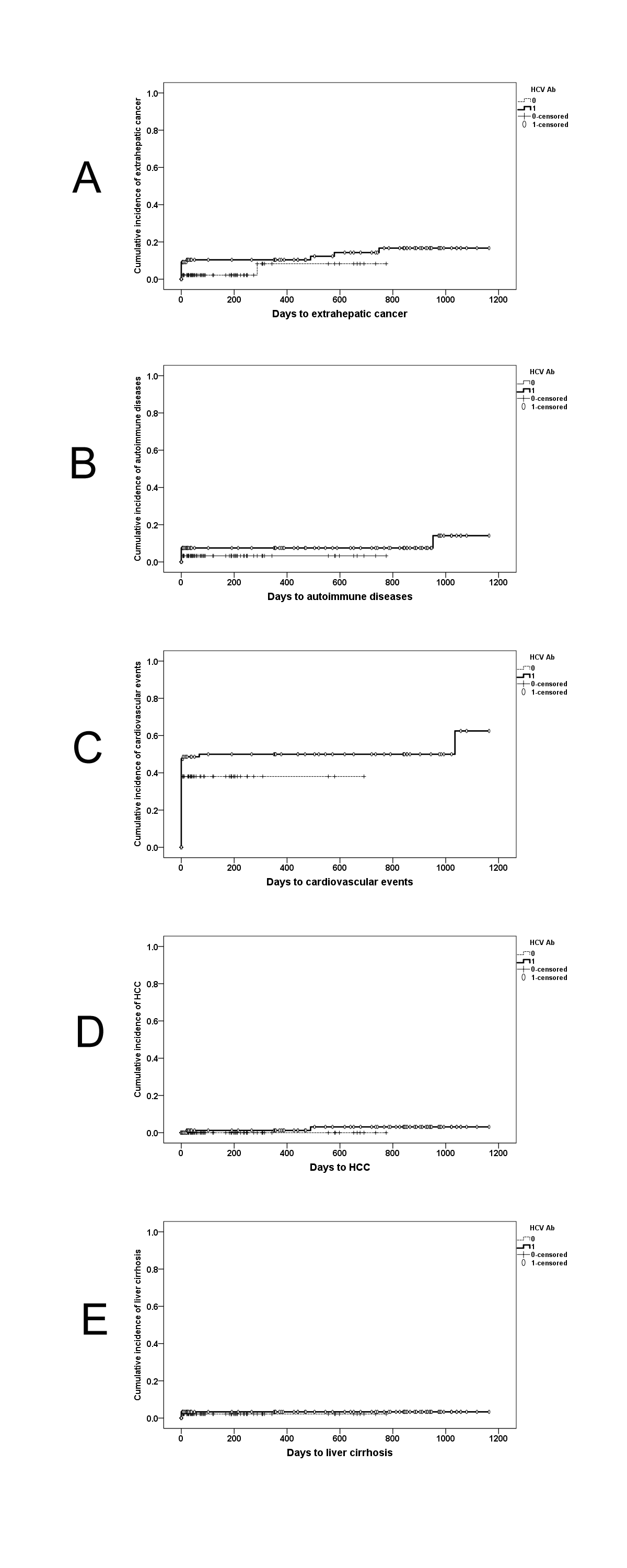

Supplement: Figure S3 — Cumulative incidences of various outcomes of all patients. [file spectrum.02922-24-s0003.tif]
