# Supplementary material for: HCV Ab titer and ALT level indicate occult hepatitis C virus infection in treatment-naive HCV Ab-positive and HCV Ab-negative patients: a 3-year prospective cohort study
Source: Microbiol Spectr. 2025 Jun 24;13(8):e02922-24. doi: 10.1128/spectrum.02922-24 (PMC12323646; doi:10.1128/spectrum.02922-24)
Supplement: Supplemental legends — Legends for Fig. S1 to S3. [file spectrum.02922-24-s0004.docx]

**Supplementary Figure 1. Cumulative incidences of cardiovascular events (A), autoimmune diseases (B), extrahepatic cancer (C), liver cirrhosis (D), HCC (E) and mortality (F) among HCV Ab-positive patients with** (solid line) **and without** (dashed line) **OCI.**

**Supplementary Figure 2. Cumulative incidences of autoimmune diseases (A), cardiovascular events (B), extrahepatic cancer (C) and liver cirrhosis (D) among HCV Ab-negative patients with** (solid line) **and without** (dashed line) **OCI.**

**Supplementary Figure 3. Cumulative incidences of extrahepatic cancer (A), autoimmune diseases (B), cardiovascular events (C), HCC (D) and liver cirrhosis (E) among HCV Ab-positive** (solid line) **and HCV-negative** (dashed line) patients**.**
