# Supplementary material for: HCV Ab titer and ALT level indicate occult hepatitis C virus infection in treatment-naive HCV Ab-positive and HCV Ab-negative patients: a 3-year prospective cohort study
Source: Microbiol Spectr. 2025 Jun 24;13(8):e02922-24. doi: 10.1128/spectrum.02922-24 (PMC12323646; doi:10.1128/spectrum.02922-24)
Supplement: Table S1 — Extrahepatic cancers of HCV Ab-positive patients. [file spectrum.02922-24-s0005.docx]

**Supplementary Table 1. Extrahepatic cancers of HCV Ab-positive patients (n=128)**

|  | **OCI (+) (n=15)** | **OCI (-) (n=113)** |
| --- | --- | --- |
| Breast Cancer, n (%) | 1 (6.6) | 1 (0.8) |
| Colon cancer, n (%) |  | 3 (2.6) |
| Larynx cancer, n (%) |  | 1 (0.8) |
| Lung cancer, n (%) |  | 2 (1.7) |
| Lymphoma, n (%) |  | 1 (0.8) |
| Pancreas cancer, n (%) |  | 1 (0.8) |
| Prostate cancer, n (%) |  | 2 (1.7) |
| Stomach, n (%) |  | 1 (0.8) |

OCI: occult hepatitis C virus infection
